# Long noncoding RNA CCDC183-AS1 depletion represses breast cancer cell proliferation, colony formation, and motility by sponging microRNA-3918

**DOI:** 10.32604/or.2022.03573

**Published:** 2022-08-01

**Authors:** TAO LIU, LIMIN ZHOU, LIANBO ZHANG, XIN GUAN, YI DONG

**Affiliations:** 1 Department of Second Breast Surgery, Jilin Cancer Hospital, Changchun, 130012, China; 2 Medical Insurance Guarantee Office, Jilin Cancer Hospital, Changchun, 130012, China; 3 Department of Breast Surgery, The First Hospital of Jilin University, Changchun, 130061, China

**Keywords:** Long noncoding RNA, CCDC183-AS1, microRNA, ceRNA

## Abstract

Many studies have illustrated the significance of long noncoding RNAs in oncogenesis and promotion of breast cancer (BC). However, the biological roles of CCDC183 antisense RNA 1 (CCDC183-AS1) in BC have rarely been characterized. Thus, we explored whether CCDC183-AS1 is involved in the malignancy of BC and elucidated the possible underlying mechanisms. Our data confirmed elevated CCDC183-AS1 expression in BC, which was associated with poor clinical outcomes. Functionally, knocking down CCDC183-AS1 hampered cell proliferation, colony formation, migration, and invasion in BC. Additionally, the absence of CCDC183-AS1 restrained tumor growth *in vivo*. Mechanistically, CCDC183-AS1 executed as a competitive endogenous RNA in BC cells by decoying microRNA-3918 (miR-3918) and consequently overexpressing fibroblast growth factor receptor 1 (FGFR1). Furthermore, functional rescue experiments confirmed that inactivation of the miR-3918/FGFR1 regulatory axis by inhibiting miR-3918 or increasing FGFR1 expression could abrogate the CCDC183-AS1 ablation-mediated repressive effects in BC cells. In summary, CCDC183-AS1 deteriorates the malignancy of BC cells by controlling miR-3918/FGFR1 regulatory axis. We believe that our study can deepen our understanding of BC etiology and contribute to an improvement in treatment choices.

## Introduction

Around the world, breast cancer (BC) is the most common cancer among women, accounting for approximately one-fourth of tumor cases [1]. Globally, deaths due to BC rank second in women [2]. At present, surgery together with chemotherapy and radiotherapy is the first-line treatment option for BC [3]. Despite substantial development of diagnostic and therapeutic technology, the clinical outcomes of patients with BC remain dismal due to the high aggressiveness and early metastasis of BC [4]. The 5-year survival rate of patients with BC with distant metastasis is only 26% [5]. Another reason for the unsatisfactory prognosis is that the molecular mechanisms underlying BC pathogenesis are not fully understood [6]. Thus, identifying novel therapies to address the malignancy of BC is of significant importance to improve the prognosis of BC.

Long noncoding RNAs (lncRNAs) are a novel category of noncoding RNA transcripts harboring over 200 nucleotides. Although lncRNAs exert no protein-coding function, they are extensively implicated in gene expression modulation, chromosome remodeling, and other physiological and pathological processes [7,8]. In recent years, the detailed roles of lncRNAs in BC have received increasing interest and are being heavily studied [9–11]. The aberrant expression of lncRNAs is frequently observed in BC, implying that they may take part in the malignancy of BC [12–14]. Accumulating evidence has highlighted the antimalignancy or carcinogenic activities of lncRNAs, thus implicating their important regulatory roles in BC [15]. LncRNAs and microRNAs (miRNAs) can interact with each other, thus affecting the metabolic activities of cells [16,17]. In the competitive endogenous RNA (ceRNA) theory, lncRNAs harbor miRNA response elements and can competitively bind to miRNAs, thereby weakening the repressive effect of miRNAs on target mRNAs and regulating genes at the posttranscriptional level [18]. Therefore, the exploration of lncRNAs in BC may be vital for the discovery of novel diagnostic and therapeutic targets.

Over 95,000 lncRNAs have been verified in the human genome [19], yet few lncRNAs have been investigated in depth in BC. Firstly, using the TCGA database, we found that CCDC183-AS1 was one of the most overexpressed lncRNAs in BC. Therefore, we chose CCDC183-AS1 as the study object of our present study. Here, we studied CCDC183-AS1 in BC, which was previously uncharacterized. We then explored its detailed roles and unveiled the possible underlying mechanisms. Our study revealed a novel ceRNA regulatory pathway involving CCDC183-AS1, miR-3918, and FGFR1 that participates in the oncogenesis and progression of BC.

## Materials and Methods

### Tissue samples

This study was approved by the Research Ethics Committee of Jilin Cancer Hospital. Written informed consent forms were signed by all participants. Human BC tissues were obtained from 56 patients with BC who were admitted to our hospital. Adjacent normal tissues were obtained at least 3 cm away from tumor tissues. The exclusion criteria were as follows: patients suffering from other human cancer types, patients who had undergone radiotherapy or chemotherapy, patients with severe diseases of the blood system, and patients who declined to enroll in the research study.

### Cell lines

The human immortalized breast epithelial cell line MCF-10A (ATCC, Manassas, VA, USA) was grown in MEGM^™^ Mammary Epithelial Cell Growth Medium BulletKit^™^ (Lonza/Clonetics Corporation, Walkersville, MD, USA) containing 100 ng/ml cholera toxin. BC cell lines MDA-MB-231 (ATCC) and MDA-MB-468 (Chinese Academy of Medical Sciences; Shanghai, China) were cultured in L-15 medium (Gibco; Thermo Fisher Scientific, Inc., Waltham, MA, USA), while McCoy’s 5a medium (Gibco; Thermo Fisher Scientific, Inc.) was applied for maintaining BC cell line SK-BR-3 (ATCC). BC cell line MCF-7 (Chinese Academy of Medical Sciences) was cultured in minimum essential medium containing 1% Glutamax, 1% nonessential amino acids, 1% sodium pyruvate 100-mM solution (all from Gibco; Thermo Fisher Scientific, Inc.), and human recombinant insulin (Sigma-Aldrich, St. Louis, MO, USA). Meanwhile, 10% fetal bovine serum (FBS) and 1% penicillin/streptomycin (Gibco; Thermo Fisher Scientific, Inc.) were added in all culture media. All cell lines were grown at 37°C in saturated humidity with 5% CO_2_.

### Transfection

The sequences of three specific siRNAs targeting CCDC183-AS1 (si-CCDC183-AS1) were acquired from GenePharma (Shanghai, China). Negative control (NC) siRNA (si-NC) served as the control of si-CCDC183-AS1. The miR-3918 mimic and miR-3918 inhibitor (anti-miR-3918) were purchased from Sangon Biotech (Shanghai, China) and used to alter endogenous miR-3918 expression. NC mimic and NC inhibitor (anti-NC) acted as the transfection reference. To increase FGFR1 expression, pcDNA3.1-FGFR1 (pc-FGFR1) was produced by GenePharma. The above molecular products were transfected into BC cells applying Lipofectamine® 2000 (Invitrogen, Carlsbad, CA, USA). Approximately 6 h after transfection, the culture medium was removed, and a fresh culture medium was added.

### RNA preparations and quantitative reverse transcription-polymerase chain reaction

The isolation of total RNA from collected samples or cells was achieved using a total RNA Purification Kit (Norgen Biotek Corp., Belmont, CA, USA). For CCDC183-AS1 and FGFR1 quantification, first-strand complementary DNA was prepared using a PrimeScript^™^ RT Reagent kit with gDNA Eraser (Takara, Tokyo, Japan). PCR amplification was then implemented using TB Green Premix Ex Taq (Takara). GAPDH was used as the control for si-CCDC183-AS1 and FGFR1.

RNAiso for Small RNA (Takara) was used to isolate small RNA. Small RNA was reverse transcribed into complementary DNA employing a miScript Reverse Transcription Kit (Qiagen, Hilden, Germany). Next, the miScript SYBR Green PCR Kit (Qiagen) was used to determine miR-3918 expression. U6 served as the reference for miRNA. The expression of all genes was analyzed using the 2^−ΔΔCq^ method.

### Cell counting kit-8 assay

Transfected cells were collected and diluted in a complete culture medium at a density of 2 × 10^4^ cells per ml. Every well of a 96-well plate was filled with 100 µl of cell suspension, while cell cultivation was conducted at 37°C for different periods. Cell proliferation was monitored by treatment with 10 µl of cell counting kit-8 (CCK-8) reagent (Dojindo, Kumamoto, Japan) at 37°C for 2 h. The optical density values at 450-nm (OD 450) wavelength were measured using a microplate reader.

### Colony formation analysis

At 24-h posttransfection, BC cells were treated with 0.25% trypsin, and the single-cell suspension was obtained. Transfected cells were seeded into 6-well plates with a density of 5000 cells per well. The colonies were stained at 37°C with 0.01% crystal violet after cultivation for 14 days. Next, the counting of newly formed colonies was implemented under an inverted light microscope (Olympus; Tokyo, Japan).

### Transwell migration and invasion assays

For the invasion assay, the upper surface of the Transwell insert was precoated with Matrigel (BD Biosciences, Franklin Lakes, NJ, USA). This step was not required for the migration test. After trypsinization, the transfected cells were resuspended in a culture medium without FBS. The upper chambers were covered with 100 µl of cell suspension containing 1 × 10^5^ cells. A volume of 600 µl of 20% FBS-supplemented culture medium had chemotactic activity and was added to the bottom chambers. After 24 h of cultivation, the migrated or invaded cells were fixed with 100% methanol, subsequently stained with 0.1% crystal violet, and then photographed (×200 magnification).

### Tumor xenograft assay

Special sequences of short-hairpin RNA (shRNA) targeting CCDC183-AS1 (sh-CCDC183-AS1) were designed and obtained from GenePharma. The shRNAs were subcloned into the pLKO.1 plasmid and transfected into 293T cells in parallel with the psPAX2 and pMD2.G plasmid, yielding lentiviruses overexpressing sh-CCDC183-AS1 or sh-NC. SK-BR-3 cells were infected with the appropriate lentivirus and treated with puromycin to chosen stable CCDC183-AS1-depleted cells.

The animal experiment was approved by the Institutional Animal Care and Use Committee of Jilin Cancer Hospital. Female BALB/c nude mice, aged 4–6 weeks old, were acquired from Beijing Vital River Laboratory Animal Technology Co., Ltd., (Beijing, China). Stable sh-CCDC183-AS1- or sh-NC-transfected CCDC183-AS1 MCF-7 cells (2 × 10^6^) were subcutaneously inoculated into the flanks of nude mice. Tumor size was observed every 4 days and applied for the calculation of tumor volume using the following equation: tumor volume (mm^3^) = 0.5 × (length × width^2^). Four weeks after tumor cell injection, all mice were euthanized by cervical dislocation, and tumor xenografts were resected and weighed.

### Subcellular fractionation assay

A nuclear/cytosol fractionation kit (BioVision, CA, USA) was adopted as a system that enabled the separation of nuclear extract from the cytoplasmic fraction of BC cells. A reverse transcription-polymerase chain reaction (qRT-PCR) was carried out to examine CCDC183-AS1 expression in both fractions after RNA separation from the nuclear/cytoplasmic fractions.

### Bioinformatic analysis

StarBase 3.0 (http://starbase.sysu.edu.cn/) and miRDB (http://mirdb.org/) were used to predict the binding between CCDC183-AS1 and miR-3918. TargetScan (http://www.targetscan.org/) and StarBase 3.0 were employed to forecast the possible target genes of miR-3918.

### Luciferase reporter assay

The CCDC183-AS1 and FGFR1 sequences containing the wild-type (Wt) miR-3918 binding site were synthesized by GenePharma and inserted into the pmirGLO reporter plasmid, represented as CCDC183-AS1-Wt and FGFR1-Wt, respectively. The same experimental steps were employed to obtain the mutant (Mut) reporter plasmids, namely, CCDC183-AS1-Mut and FGFR1-Mut. Using Lipofectamine® 2000, BC cells were transfected with the Wt or corresponding Mut reporter plasmid alongside miR-3918/NC mimic. After 2 days, a dual-luciferase reporter assay system (Promega, Madison, WI, USA) was applied for luciferase activity measurement.

### RNA immunoprecipitation (RIP)

Magna RIP^™^ RNA-binding protein immunoprecipitation kit (Millipore, Billerica, MA, USA) was used in the experiment. Briefly, BC cells were lysed in RIP cell lysis buffer, and the whole-cell extract was collected and cultivated overnight at 4°C with RIP buffer that was supplemented with magnetic beads conjugated with human Ago2 or IgG antibody (Millipore). Thereafter, the beads were harvested and treated with Protease K to digest the protein. The immunoprecipitated RNA was isolated and examined with qRT-PCR.

### Western blotting

Tissue specimens or cells were lysed using radio-immunoprecipitation assay lysis buffer (Solarbio, Beijing, China). A BCA Kit (Solarbio) was used to achieve the quantification of total protein. Equivalent proteins were loaded and isolated by employing 10% sodium dodecyl sulfate polyacrylamide gel electrophoresis. After being transferred onto a polyvinylidene fluoride membrane, 5% skimmed milk was used to block the nonspecific binding sites. The membranes were then added with primary antibodies targeting FGFR1 (ab76464) or GAPDH (ab181602; Abcam, Cambridge, UK) and cultivated at 4°C for 12 h, followed by extensive washing with Tris-buffered saline-tween three times and treatment with a secondary antibody (ab6721; Abcam). The protein bands were detected by applying Pierce^™^ ECL western blotting substrate (Pierce).

### Statistical analysis

The SPSS 18.0 software package (IBM Corp., Armonk, NY, USA) was employed for all statistical analyses. All experiments were repeated at least three times, and collected data are presented as the mean ± SD. The data between the two groups was compared using the Student’s *t*-test. One-way analysis of variance together with Tukey’s test was adopted to test the differences among multiple groups. A *P* value of <0.05 indicates a significant difference.

## Results

### CCDC183-AS1 is overexpressed in breast cancer and exerts carcinogenic roles

**FIGURE 1:**
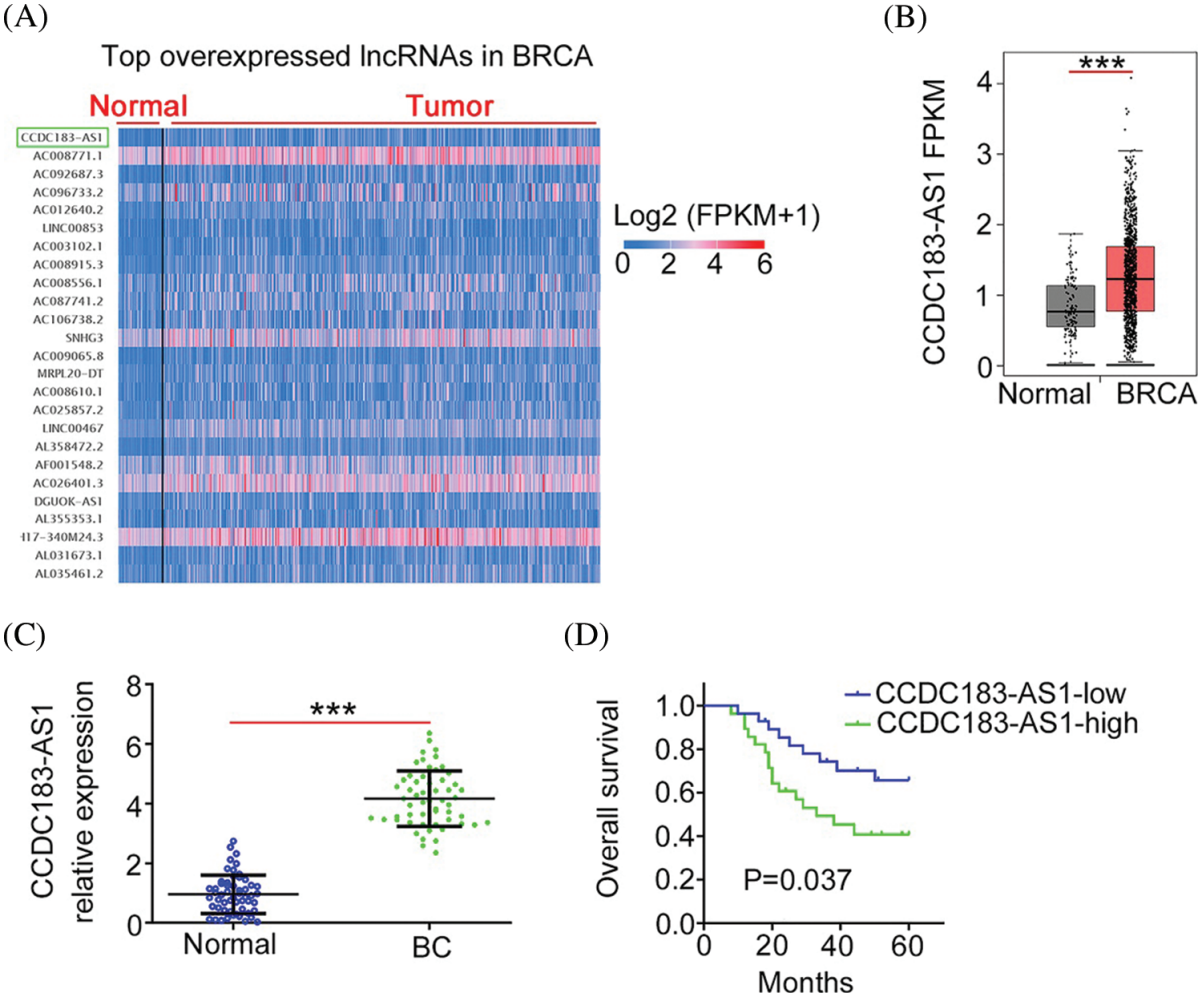
CCDC183-AS1 was overexpressed in BC. (A) CCDC183-AS1 was the 76^th^ overexpressed lncRNA in BRCA. (B) The TCGA database was used to analyze the CCDC183-AS1 expression in sarcoma. ***p < 0.001 *vs.* normal. (C) CCDC183-AS1 expression in BC tissues was examined by applying qRT-PCR. ***p < 0.001 *vs.* normal. (D) The overall survival of patients with BC presenting high or low CCDC183-AS1 levels was tested using the Kaplan–Meier analysis. *p* = 0.037.

First, using the TCGA database, we found that CCDC183-AS1 (Fig. 1A) was the 76^th^ overexpressed lncRNA in breast invasive carcinoma (BRCA). The level of CCDC183-AS1 was higher in BRCA than in normal tissues (Fig. 1B). Next, 56 BC tissues were collected and matched adjacent normal tissues were taken as controls. Compared to adjacent normal tissues, CCDC183-AS1 was apparently upregulated in BC tissues (Fig. 1C). The median value of CCDC183-AS1 in BC tissues was defined as the cutoff value, and all 56 BC patients were divided into either CCDC183-AS1-low or CCDC183-AS1-high groups. Patients with BC characterized by a high CCDC183-AS1 level had evidently shorter overall survival compared with patients characterized by a low CCDC183-AS1 level (Fig. 1D).

**FIGURE 2:**
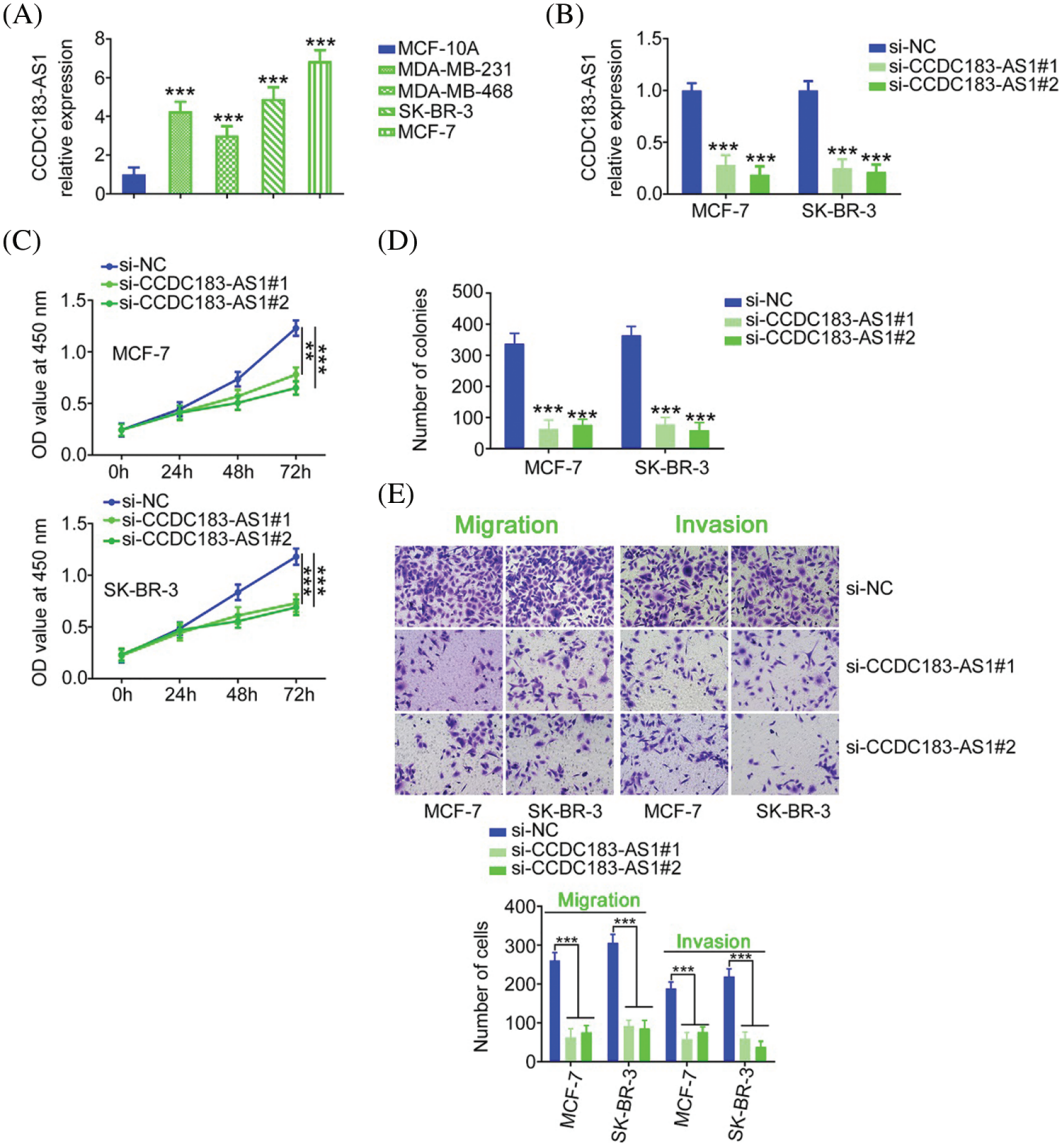
CCDC183-AS1 downregulation restricts the aggressiveness of BC cells. (A) The expression pattern of CCDC183-AS1 in BC cell lines. ***p < 0.001 (n = 3) *vs*. MCF-7. (B) Two siRNAs targeting CCDC183-AS1 were delivered into BC cells. The efficiency of si-CCDC183-AS1 transfection was detected by qRT-PCR. ***p < 0.001 (n = 3) *vs*. si-NC. (C) CCK-8 assay examined the proliferative ability of BC cells when CCDC183-AS1 was knocked down. ***p < 0.001 (n = 3) *vs*. si-NC. **p < 0.01 (n = 3) *vs*. si-NC. (D) Colony formation in CCDC183-AS1-silenced BC cells. ***p < 0.001 (n = 3) *vs*. si-NC. (E) Migration and invasion (×200 magnification) of BC cells after CCDC183-AS1 deficiency. ***p < 0.001 (n = 3) *vs*. si-NC.

To unveil the role of CCDC183-AS1 in BC malignancy, CCDC183-AS1 was silenced in BC cells. Before that step, qRT-PCR was implemented to detect CCDC183-AS1 expression in BC cell lines. Compared with MCF-10A, the level of CCDC183-AS1 was distinctly increased in the tested BC cell lines (Fig. 2A). Cell lines MCF-7 and SK-BR-3 presented the highest levels among the four BC cell lines; thus, they were selected for functional experiments. The siRNAs targeting CCDC183-AS1 were transfected into the MCF-7 and SK-BR-3 cells. qRT-PCR analysis verified the interference efficiency, demonstrated by the decreased level of CCDC183-AS1 in BC cell lines (Fig. 2B). Two siRNAs were used in the following experiments to avoid off-target effects. Through the CCK-8 assay, the data manifested the decreased proliferative capacity of BC cells after CCDC183-AS1 depletion (Fig. 2C). Additionally, cell colony formation was clearly impaired following CCDC183-AS1 ablation (Fig. 2D). Furthermore, knocking down CCDC183-AS1 weakened BC cell migration and invasion (Fig. 2E). Thus, CCDC183-AS1 is an oncogenic lncRNA in BC.

### CCDC183-AS1 acts as a miR-3918 sponge in breast cancer

**Figure 3:**
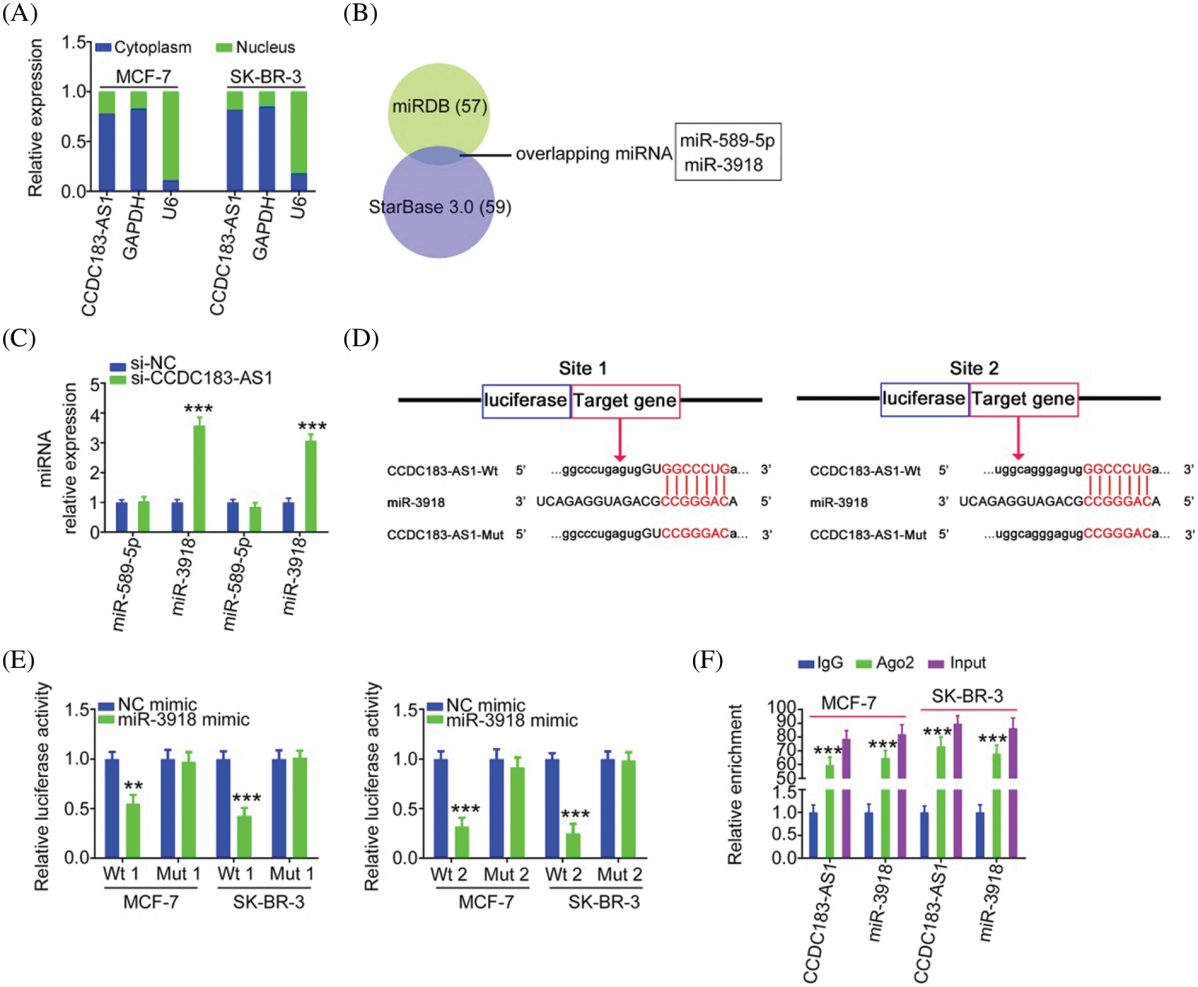
CCDC183-AS1 sponges miR-3918 in BC. (A) Location of CCDC183-AS1 in BC cells. (B) Two overlapping miRNAs interacting with CCDC183-AS1 were searched by miRDB and StarBase 3.0. (C) Expression of miR-589-5p and miR-3918 in BC cells with CCDC183-AS1 depletion. ***p < 0.001 (n = 3) *vs*. si-NC. (D) The predicted binding sites of miR-3918 within CCDC183-AS1 and mutant binding sites are shown. (E) Luciferase reporter assays applying BC cells cotransfected with miR-3918/NC mimic and CCDC183-AS1-Wt or CCDC183-AS1-Mut. ***p < 0.001 (n = 3) *vs*. NC mimic. **p < 0.01 (n = 3) *vs*. NC mimic. (F) RIP assay affirmed that CCDC183-AS1 and miR-3918 were enriched by the Ago2 antibody in BC cells. ***p < 0.001 (n = 3) *vs*. IgG.

Having identified the oncogenic actions of CCDC183-AS1 in BC, we uncovered the downstream mechanisms of CCDC183-AS1. Initially, a subcellular fractionation assay was performed to assess the location of CCDC183-AS1, which was found to be mostly distributed in BC cell cytoplasm (Fig. 3A). The outcomes implied that CCDC183-AS1 may act as a miRNA sponge or ceRNA, thereby activating posttranscriptional modification. To confirm our hypothesis, bioinformatic analysis was completed applying StarBase 3.0 and miRDB, and two overlapping miRNAs (miR-589-5p and miR-3918) harboring complementary sequences within CCDC183-AS1 were screened out (Fig. 3B). The miR-3918 level was increased in BC cells after CCDC183-AS1 deficiency, whereas the amount of miR-589-5p was unaltered following si-CCDC183-AS1 transfection (Fig. 3C). Accordingly, we presumed that miR-3918 may be a downstream target of CCDC183-AS1 (Fig. 3D).

For validation, a luciferase reporter assay was executed. The results illustrated that introduction of the miR-3918 mimic contributed to an obvious downregulation of the luciferase activity of CCDC183-AS1-Wt (Fig. 3E). However, the luciferase activity could not be modulated by miR-3918 in BC cells upon mutation of the binding sequences, suggesting direct binding between miR-3918 and CCDC183-AS1. The RIP assay then illustrated that miR-3918 and CCDC183-AS1 were remarkably enriched by the Ago2 antibody in BC cells (Fig. 3F). Collectively, CCDC183-AS1 acts as a natural molecular sponge of miR-3918 in BC.

### Lowering miR-3918 expression reverses the inhibitory effects on BC cell proliferation, colony formation, and motility triggered by CCDC183-AS1 knockdown

**FIGURE 4:**
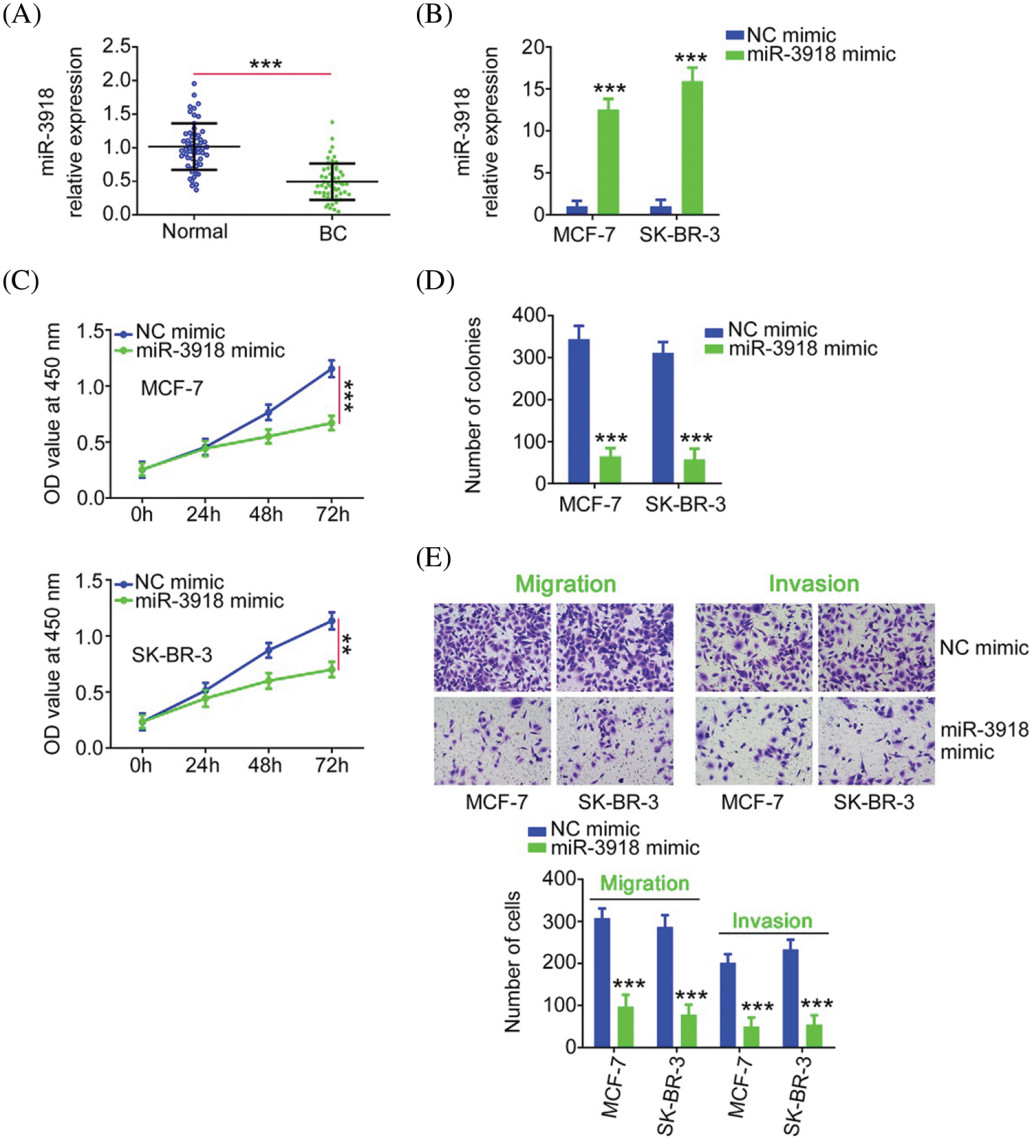
miR-3918 harms the malignancy of BC. (A) miR-3918 expression in BC tissues. ***p < 0.001 (n = 3) *vs*. normal. (B) The transfection efficiency of the miR-3918 mimic was tested in BC cells. ***p < 0.001 (n = 3) *vs*. NC mimic. (C, D) The proliferation and colony formation of miR-3918-overexpressed BC cells were examined. **p < 0.01 (n = 3) *vs*. NC mimic. ***p < 0.001 (n = 3) *vs*. NC mimic. (E) The influences of miR-3918 upregulation on BC cell migration (×200 magnification) and invasion (×200 magnification) were uncovered. ***p < 0.001 (n = 3) *vs*. NC mimic.

To explore the physiological roles of miR-3918 in BC, we firstly detected its expression status in BC, and qRT-PCR analysis presented poor miR-3918 expression in BC tissues (Fig. 4A). The miR-3918 expression was overexpressed in BC cells by transfection with a miR-3918 mimic (Fig. 4B). Overexpressed miR-3918 dampened cell proliferation and colony formation (Figs. 4C and 4D) while weakening cell migration and invasion (Fig. 4E) in BC.

**FIGURE 5:**
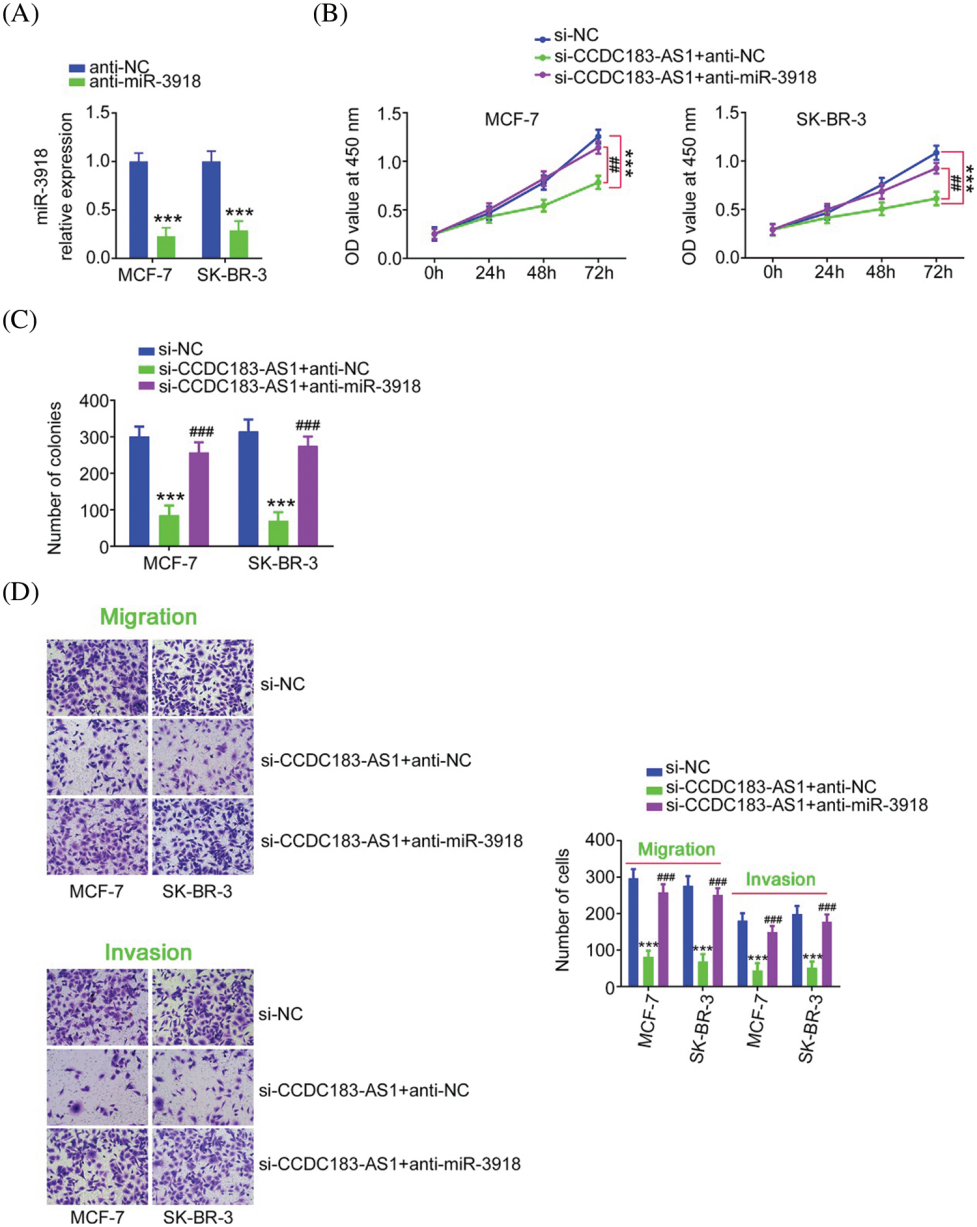
miR-3918 downregulation could offset the impacts of CCDC183-AS1 upregulation on BC cells. (A) The level of miR-3918 in anti-miR-3918-transfected BC cells was quantified via qRT-PCR. ***p < 0.001 (n = 3) *vs*. anti-NC. (B–D) CCDC183-AS1-silenced BC cells underwent cotransfection of anti-miR-3918 or anti-NC and were subjected to the evaluation of cell proliferation, colony formation, migration (×200 magnification), and invasion (×200 magnification). ***p < 0.001 (n = 3) *vs*. anti-NC. ^##^*p* < 0.01 (n = 3) *vs*. si-CCDC183-AS1+anti-NC. ^###^*p* < 0.001 (n = 3) *vs*. si-CCDC183-AS1+anti-NC.

Rescue experiments were implemented to illustrate whether the cancer-inhibiting roles played by si-CCDC183-AS1 in BC cells were achieved by targeting miR-3918. Before that, qRT-PCR demonstrated the knockdown efficiency of anti-miR-3918 in BC cells (Fig. 5A). Interference with CCDC183-AS1 suppressed BC cell proliferation. Conversely, inhibition of miR-3918 prevented this inhibition (Fig. 5B). Furthermore, anti-miR-3918 alleviated the regulatory effects of CCDC183-AS1 depletion on colony formation (Fig. 5C), migration, and invasion (Fig. 5D) of BC cells.

### FGFR1, a downstream target of miR-3918, is controlled by CCDC183-AS1 in breast cancer

**FIGURE 6:**
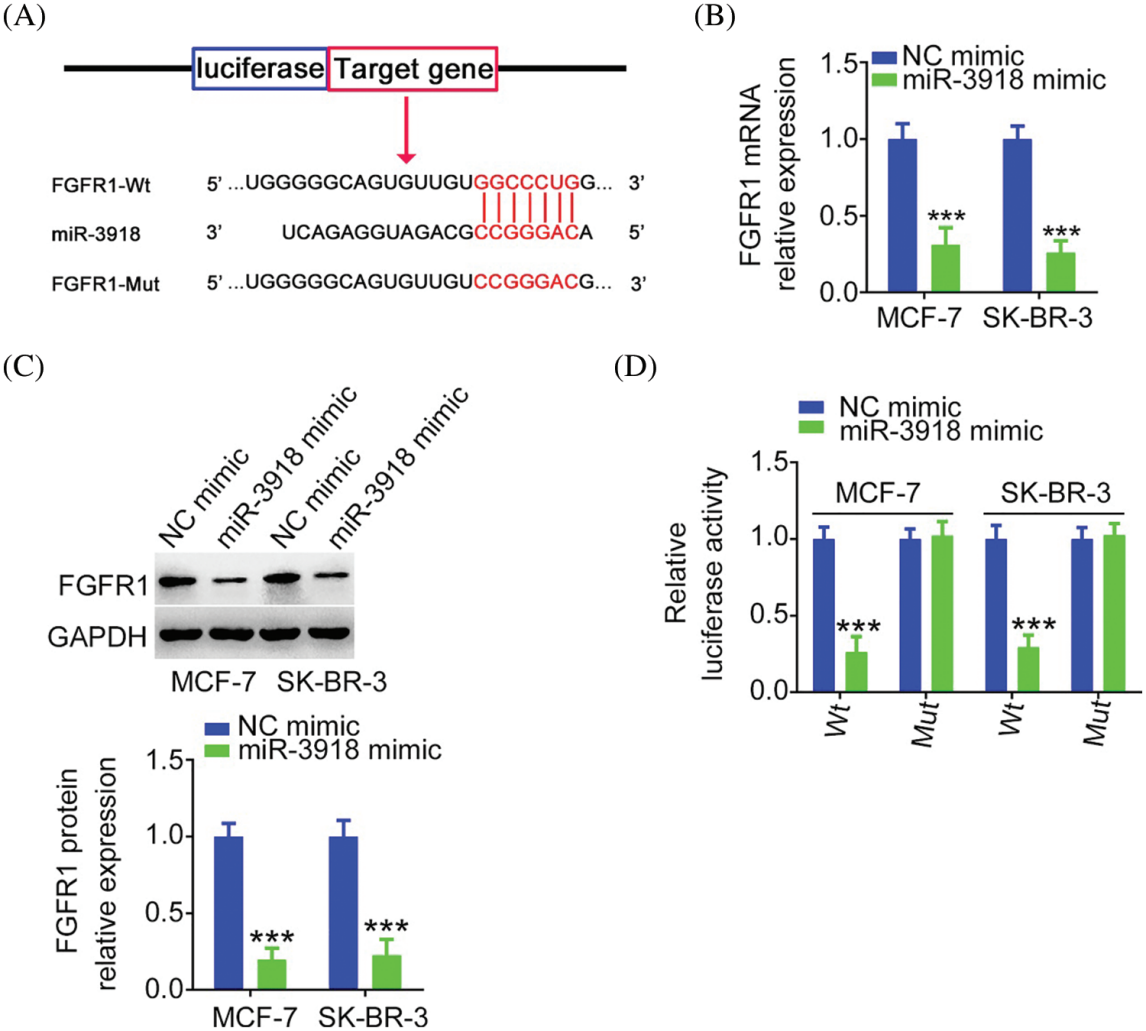
FGFR1 is a direct target of miR-3918 in BC. (A) The complementary wild-type and mutant binding sequences between miR-3918 and FGFR1 3′-UTR. (B, C) FGFR1 mRNA and protein expression in miR-3918-overexpressed BC cells. ***p < 0.001 (n = 3) *vs*. NC mimic. (D) Luciferase reporter assays using BC cells cotransfected with miR-3918/NC mimic and FGFR1-Wt or FGFR1-Mut. ***p < 0.001 (n = 3) *vs*. NC mimic.

miRNAs work by directly binding to the 3′-UTR of target genes and then negatively regulating them. The putative target of miR-3918 was predicted *via* bioinformatic tools. FGFR1 was predicted as a potential target of miR-3918 (Fig. 6A) and was chosen for following mechanistic investigations. By detecting BC cells after miR-3918 mimic transfection, FGFR1 expression (Figs. 6B and 6C) was strikingly suppressed by miR-3918 upregulation. The luciferase reporter assay results showed that miR-3918 overexpression evidently suppressed the luciferase activity of FGFR1-Wt in BC cells, but it had no impact on the luciferase activity of FGFR1-Mut (Fig. 6D).

**FIGURE 7:**
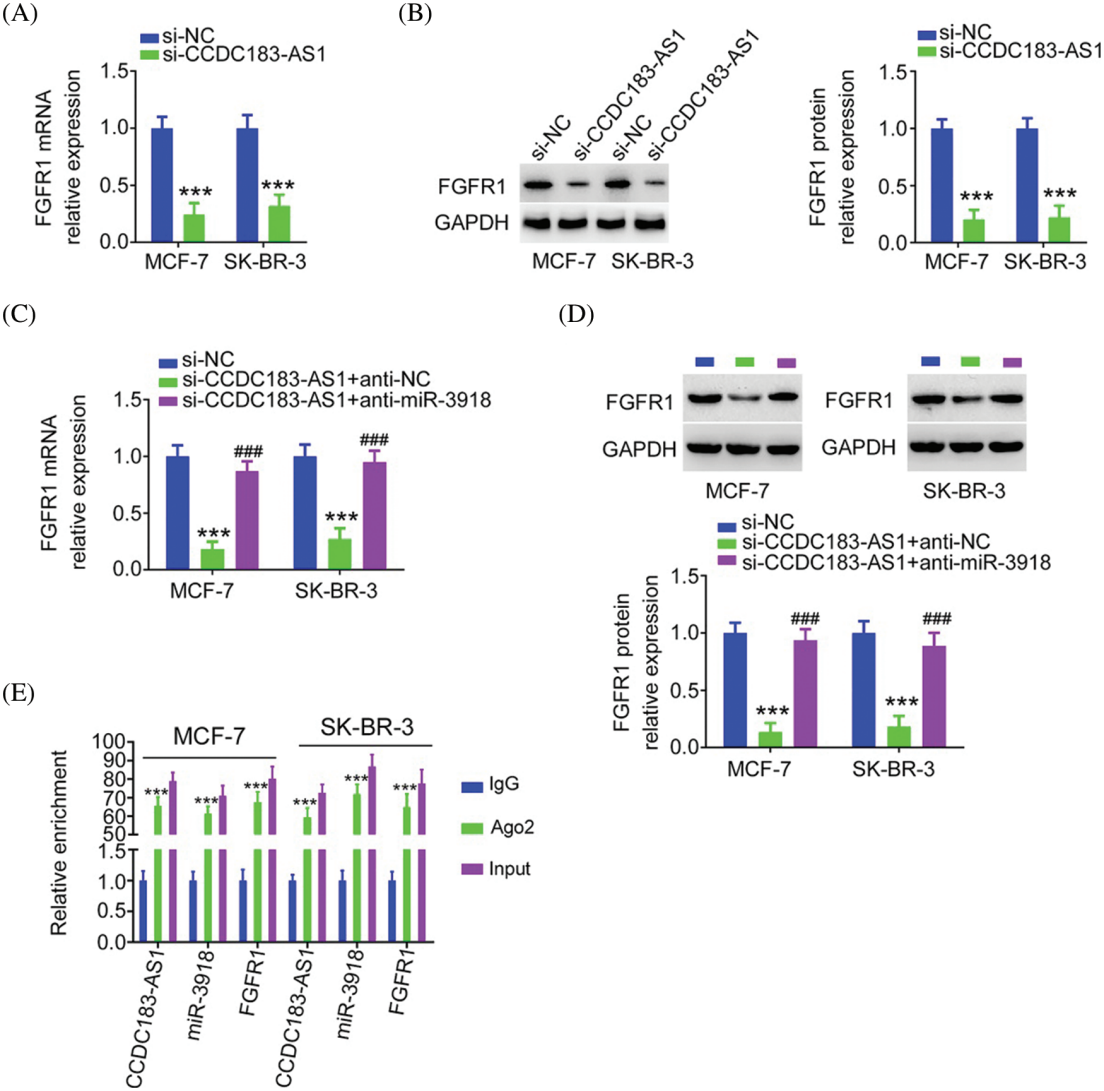
FGFR1 is controlled by CCDC183-AS1/miR-3918 axis in BC. (A, B) FGFR1 mRNA and protein expression in si-CCDC183-AS1-transfected BC cells. ***p < 0.001 (n = 3) *vs*. si-NC. (C, D) BC cells were transfected with anti-miR-3918 or anti-NC together with si-CCDC183-AS1 and subjected to the measurement of FGFR1 expression. ***p < 0.001 (n = 3) *vs*. si-NC. ^###^*p* < 0.001 (n = 3) *vs*. si-CCDC183-AS1 + anti-NC. (E) RIP assay affirmed that CCDC183-AS1, miR-3918, and FGFR1 were enriched by the Ago2 antibody in BC cells. ***p < 0.001 (n = 3) *vs*. IgG.

Next, as miR-3918 directly targeted FGFR1, we wanted to explore whether CCDC183-AS1 could modulate FGFR1 expression. The qRT-PCR and western blotting data supported our hypothesis, as FGFR1 expression (Figs. 7A and 7B) was obviously decreased in si-CCDC183-AS1-transfected BC cells, which was counteracted by anti-miR-3918 treatment (Figs. 7C and 7D). Besides, CCDC183-AS1, miR-3918, and FGFR1 were all notably enriched by the Ago2 antibody in BC cells (Fig. 7E). Altogether, FGFR1 expression is positively regulated by CCDC183-AS1/miR-3918 in a ceRNA-dependent manner.

### FGFR1 reintroduction is capable of abolishing the repressing action of CCDC183-AS1 knockdown on BC cell proliferation, colony formation, and motility

**FIGURE 8:**
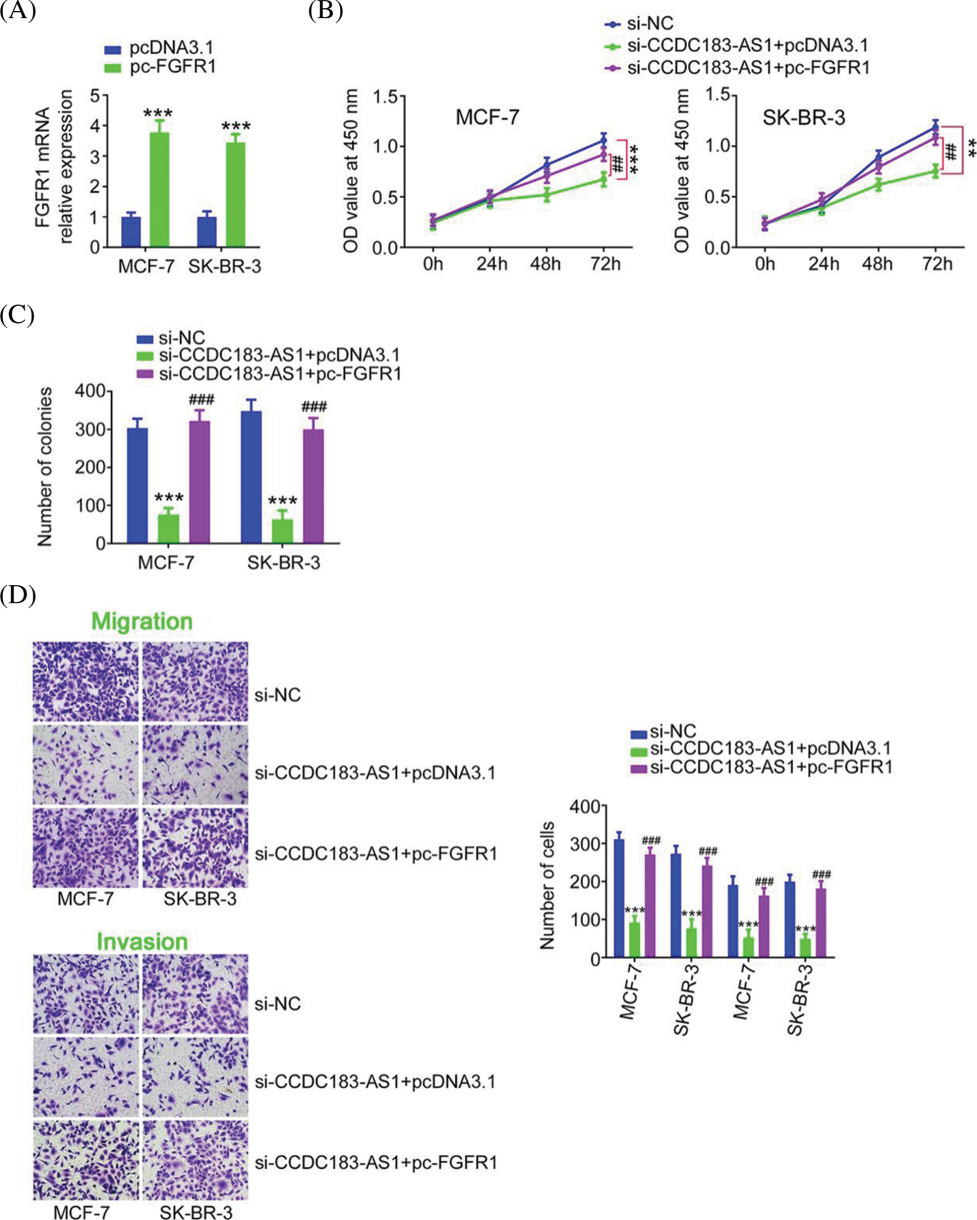
Reintroduction of FGFR1 abrogated the suppression of BC progression caused by CCDC183-AS1 ablation. (A) The efficiency of pc-FGFR1 transfection in BC cells. ***p < 0.001 (n = 3) *vs*. IgG. (B–D) The pc-FGFR1 or pcDNA3.1 plasmid was transfected into CCDC183-AS1-deficient BC cells, followed by the assessment of cell proliferation, colony formation, and motility (×200 magnification). **p < 0.01 (n = 3) *vs*. si-NC. ***p < 0.001 (n = 3) *vs*. si-NC. ^##^*p* < 0.001 (n = 3) *vs*. si-CCDC183-AS1+pcDNA3.1. ^###^*p* < 0.001 (n = 3) *vs*. si-CCDC183-AS1+pcDNA3.1.

The FGFR1 overexpression plasmid pc-FGFR1 (Fig. 8A) or empty pcDNA3.1 was transfected into BC cells in the presence of si-CCDC183-AS1. Overexpressed FGFR1 mitigated the impacts of si-CCDC183-AS1 on BC cell proliferation, colony formation, migration, and invasion (Figs. 8B–8D). Taken together, the miR-3918/FGFR1 axis was corroborated as the downstream effector of CCDC183-AS1 in BC cells.

### Downregulation of CCDC183-AS1 restrains BC tumor growth in vivo

**FIGURE 9:**
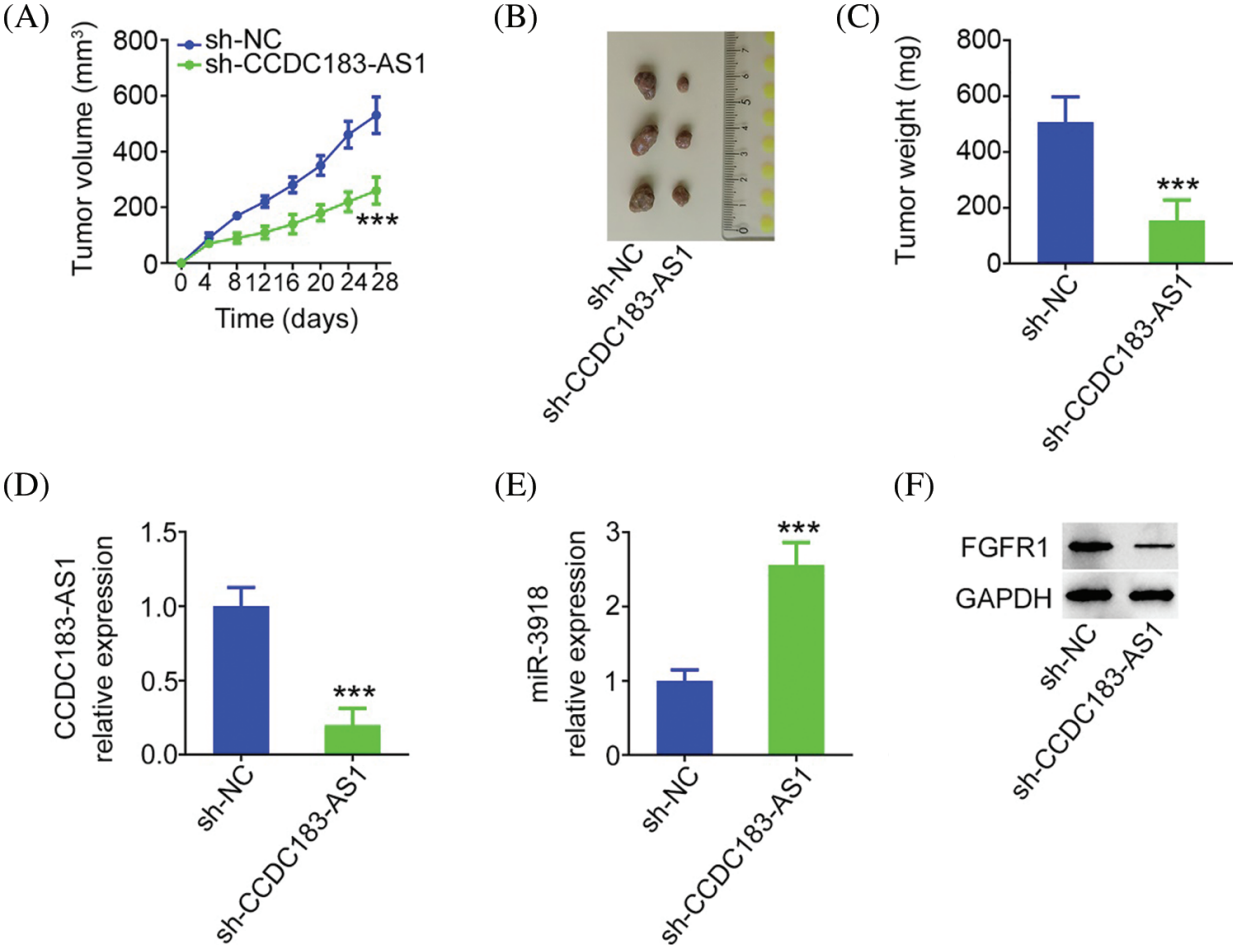
Knocking down CCDC183-AS1 hampers the *in vivo* growth of BC cells. (A) Tumor volume was monitored every 4 days. The obtained data were used in plotting growth curves. Each group contained three mice. ***p < 0.001 *vs*. sh-NC. (B) Images of tumor xenografts obtained from mice in the groups sh-CCDC183-AS1 or sh-NC. (C) Weight of the tumor xenografts. ***p < 0.001 *vs*. sh-NC. (D, E) CCDC183-AS1 and miR-3918 levels in the tumor xenografts. ***p < 0.001 *vs*. sh-NC. (F) FGFR1 protein expression in tumors.

Ultimately, whether CCDC183-AS1 affected the tumor growth *in vivo* was tested *via* tumor xenograft assay. Tumor growth of nude mice injected with sh-CCDC183-AS1 was clearly delayed in contrast to that in the sh-NC group (Fig. 9A). The tumor size and weight (Figs. 9B and 9C) were obviously reduced in the sh-CCDC183-AS1 group. Furthermore, CCDC183-AS1 was reduced, and miR-3918 was overexpressed in the tumors injected with sh-CCDC183-AS1 (Figs. 9D and 9E). Besides, as shown in Fig. 9F, FGFR1 protein levels in tumors obtained from the sh-CCDC183-AS1 group were evidently suppressed. In short, CCDC183-AS1 depletion hindered tumor growth *in vivo*.

## Discussion

Tumor metastasis and unlimited growth manifest challenges to the therapy and clinical efficiency of BC [20]. LncRNAs are deregulated in BC and contribute to the regulation of complex cellular behaviors [21–23]. Accordingly, studying the upstream regulatory mechanisms underlying BC growth and metastasis will be favorable for the development of novel pharmacological targets. Our present research discovered a novel ceRNA regulatory pathway involving CCDC183-AS1, miR-3918, and FGFR1.

To date, accumulated studies have illustrated the significance of lncRNAs in the genesis and promotion of BC. For instance, HCG18 [24], PVT1 [25], and LINC02381 [26] are highly expressed in BC and expedite tumor progression. Conversely, ADAMTS9-AS1 [27], LINC01189 [28], and ZBED3-AS1 [29] are downregulated in BC and destroy the malignancy of BC. CCDC183-AS1 was reported to be overexpressed in hepatocellular carcinoma, presenting an obvious relationship with overall survival [30]. The lncRNA performed tumor-promoting actions during hepatocellular carcinoma initiation and progression [30]. However, the biological roles of CCDC183-AS1 in BC have rarely been reported. This study confirmed elevated CCDC183-AS1 expression in BC, which was associated with poor prognosis. Functionally, knocking down CCDC183-AS1 decreased cell proliferation, colony formation, and metastasis in BC. Additionally, the absence of CCDC183-AS1 restrained tumor growth *in vivo*. Thus, CCDC183-AS1 participates in the pathogenesis of BC, and we believe that this may advance our comprehension of BC etiology and eventually the identification of new pharmacological targets.

With regard to lncRNAs, the modulation of gene expression is achieved in various ways. Gene expression in human cancer cells is mediated by lncRNAs at epigenetic transcriptional and posttranscriptional levels [31]. The mode of regulation exerted by lncRNAs is decided by their localization [32]. LncRNAs located in the cytoplasm execute posttranscriptional regulation, in which the newly discovered ceRNA is a well-studied and extensively accepted regulatory mechanism for modulating the interaction between RNA molecules *in vivo* [33]. In the field of ceRNA, lncRNAs can target the 3′-UTR of miRNAs, thereby forming the RNA-induced silencing complex and lowering the targeting effects of miRNAs on downstream effectors [32]. Therefore, subcellular fractionation experiments were conducted, and CCDC183-AS1 was identified as a cytoplasmic lncRNA. This suggests that CCDC183-AS1 may affect gene expression by functioning as a ceRNA.

Through bioinformatic analysis, we found that CCDC183-AS1 possessed a conserved binding site for miR-3918. The direct binding interaction of CCDC183-AS1 and miR-3918 was then verified by luciferase reporter assay and RIP. Interestingly, mechanistic studies certified that miR-3918 did have a repressive action on FGFR1 expression, and the latter gene could be positively regulated by CCDC183-AS1 in BC cells. In an encouraging result, rescue experiments corroborated the implication of CCDC183-AS1/miR-3918 in stimulating the expression of FGFR1. Eventually, CCDC183-AS1, miR-3918, and FGFR1 were demonstrated to be enriched by Ago2 in BC. Altogether, the three RNAs constitute a novel ceRNA pathway, i.e., CCDC183-AS1/miR-3918/FGFR1, in BC.

miR-3918 plays important roles in the aggressiveness of glioma [34] and hepatocellular carcinoma [35]; however, little is reported regarding its expression status and specific roles in BC. After observing the downregulation of miR-3918 in BC, we unveiled functions of miR-3918 using overexpression studies. Here, we affirmed that miR-3918 exerted anticarcinogenic activities in the oncogenicity of BC cells. Mechanistically, miR-3918 and the FGFR1 3′-UTR were completely complementary to each other, and miR-3918 suppressed FGFR1 expression in BC cells. Moreover, FGFR1 was positively regulated by CCDC183-AS1 in BC cells by decoying miR-3918. Functional rescue experiments confirmed that inactivation of the miR-3918/FGFR1 regulatory axis by inhibiting miR-3918 or increasing FGFR1 expression could abrogate the CCDC183-AS1 ablation-mediated suppression of BC malignancy. Therefore, the miR-3918/FGFR1 axis acts as the downstream mediator of CCDC183-AS1 in BC.

In the tumor xenograft assay, we only used three nude mice in each group. It is a limitation of our study. In future study, we will also explore the regulatory effect of CCDC183-AS1/miR-3918/FGFR1 on metastasis of BC cells *in vivo*.

In summary, our present research is the first to reveal that CCDC183-AS1 is notably upregulated in BC, indicating a poor prognosis. CCDC183-AS1 works as a ceRNA to overexpress FGFR1 by sequestering miR-3918, consequently aggravating the oncogenicity of BC cells. We believe that our study can deepen our understanding of BC etiology and contribute to an improvement in treatment choices.

## Data Availability

The datasets used and/or analyzed during the current study are available from the corresponding author on reasonable request.
